# Low Complexity Beamspace Super Resolution for DOA Estimation of Linear Array

**DOI:** 10.3390/s20082222

**Published:** 2020-04-15

**Authors:** Jie Pan, Fu Jiang

**Affiliations:** College of Information Engineering, Yangzhou University, Yangzhou 225009, China; jiangf996@163.com

**Keywords:** DOA estimation, atomic norm minimization, semidefinite programming, beamspace

## Abstract

Beamspace processing has become much attractive in recent radar and wireless communication applications, since the advantages of complexity reduction and of performance improvements in array signal processing. In this paper, we concentrate on the beamspace DOA estimation of linear array via atomic norm minimization (ANM). The existed generalized linear spectrum estimation based ANM approaches suffer from the high computational complexity for large scale array, since their complexity depends upon the number of sensors. To deal with this problem, we develop a low dimensional semidefinite programming (SDP) implementation of beamspace atomic norm minimization (BS-ANM) approach for DFT beamspace based on the super resolution theory on the semi-algebraic set. Then, a computational efficient iteration algorithm is proposed based on alternating direction method of multipliers (ADMM) approach. We develop the covariance based DOA estimation methods via BS-ANM and apply the BS-ANM based DOA estimation method to the channel estimation problem for massive MIMO systems. Simulation results demonstrate that the proposed methods exhibit the superior performance compared to the state-of-the-art counterparts.

## 1. Introduction

Direction-of-Arrive (DOA) estimation is an important topic in many applications, such as radar, sonar, and wireless communication. To lower the hardware cost, reduce the computational burden and improve the performance, the received signals on front-end sensors can be projected into lower dimensional space by digital/analog structure, which is referred to as beamspace processing [1,2,3]. In the past decades, various classic DOA estimation methods have been applied to beamspace [4,5,6,7].

Inspired from the concept of compressed sensing, some discrete sparse representation approaches are extended to the beamspace to improve the DOA estimation performance in low SNR and lack of snapshots’ scenarios [8,9,10]. However, the discrete sparse recovery methods may suffer from the off-grid sources problem, a.k.a. power leakage effect [11].

To deal with the problem, the super resolution methods have recently been proposed for gridless compressed sensing, which is referred to as atomic norm minimization (ANM) [12]. By utilizing the Toeplitz structure of the covariance matrix, ANM approaches are applied to DOA estimation of linear/rectangular arrays [13,14,15,16].

For beamspace processing cases, the existed ANM methods are usually based on the generalized line spectral estimation (GL) framework [17], which regards the beamspace DOA estimation problem as the line spectral estimation problem with linear mapping constraints, which can be solved by the conventional ANM approaches [11,18,19,20]. However, these methods focus on recovering the signal on the sensors of the receiver, which may yield high computational burden (since high dimensional SDP formulation) in the case of large number of sensors, e.g., millimeter-wave massive MIMO system for 5G, even though the dimension of the beamspace may be quite small.

Like the extensive application for gridless super resolution methods with beamspace processing in real-time scenarios, we try to propose the low complexity beamspace super resolution method that is not yet reported in the literature.

We study the beamspace super resolution approaches for discrete Fourier transform (DFT) beamspace via beamspace atomic norm minimization(BS-ANM) in this paper. Firstly, we define the beamspace atomic norm and propose the low dimensional SDP implementations based on the super resolution theory on the semi-algebraic set. Then, we develop the fast algorithm based on the alternating direction method of multipliers (ADMM) method and show the computational complexity analysis. Finally, we apply BS-ANM approaches to the covariance based DOA estimation problem and the channel estimation in millimeter-wave massive MIMO system with lens antenna array. Simulation results indicate that the proposed approaches exhibit the favorable performance and computational cost, as compared to the state-of-the-art methods.

This paper is organized as follows: the signal model and conception of atomic norm are introduced in Section 2. The low dimensional SDP implementation of BS-ANM is proposed in Section 3. In Section 4, we present a fast algorithm based on ADMM for BS-ANM and provide the complexity analysis in Section 5. In Section 6, the BS-ANM based DOA estimation and channel estimation methods are developed and simulations are performed in Section 7. Section 8 concludes this paper.

Notations: ${\left(•\right)}^{T}$ denotes the transpose, ${\left(•\right)}^{†}$ denotes pseudo inverse of a matrix, and ${\left(•\right)}^{H}$ denotes conjugate transpose of a matrix or vector. diag(x) denotes a diagonal matrix.${∥•∥}_{2}$ and ${∥•∥}_{F}$ denote the Euclidean $l_{2}$ norm and the Frobinus norm. C,R and N represent the complex, real and nature number set, respectively. M≥0 denotes positive semidifinite matrix. $E\left\{•\right\}$ is the expectation. deg(•) denotes the degree of the polynomial. vec(•) and Tr(•) are the vectorization and the trace operator. $\inf\left\{•\right\}$ and $\sup\left\{•\right\}$ denote the infimum and supremum of a set, respectively.

## 2. Signal Model and Atomic Norm

### 2.1. Signal Model

Considering the uniform linear array (ULA) of *N* sensors with the M(M<N) dimensional fixed hardware structure beamformer, there are *K* far field narrowband scatters at angular directions $\theta_{1},\ldots ,\theta_{K}$ and the measurement vector on the sensors at time *t* can be written as

(1) x(t)=As(t)+n(t),

where $s\left(t\right)={\left[s_{1},\cdots ,s_{K}\right]}^{T}\in C^{K\times 1}$ and $n\left(t\right)\in C^{N\times 1}$ are the source and additional noise component with $R_{s}=E\left\{s\left(t\right)s^{H}\left(t\right)\right\}$ and $E\left\{n\left(t\right)n^{H}\left(t\right)\right\}=\sigma^{2}I$($\sigma^{2}$ is the power of noise). The N×K array manifold A is given by

(2) $$A=[a\left(\theta_{1}\right),\cdots ,a\left(\theta_{K}\right)],$$

where the column vector of A is

(3) $$a{\left(\theta )=[1,e^{-j\frac{2\pi d}{\lambda }\sin\theta },\cdots ,e^{-j\frac{2\pi Nd}{\lambda }\sin\theta }\right]}^{T}$$

with $\theta_{k}\in \Xi$, Ξ=(−π/2,π/2], the interspace of elements *d*, and the wavelength λ.

Denote the DFT beamspace transform matrix as $W\in C^{M\times N}$, whose mth row is [4]

(4) $$w_{m}=\frac{1}{N}e^{jm\frac{(N-1)\pi }{2N}}\left[1,e^{-jm\frac{\pi }{N}},\cdots ,e^{-jm\frac{(N-1)\pi }{N}}\right],$$

the beamspace received signals are modeled as

(5) $$\begin{array}{cc} y(t) & =Bs\left(t\right)+n_{b}\left(t\right),\\ & =\sum_{k=1}^{K}s_{k}b\left(\theta_{k}\right)+n_{b}\left(t\right), \end{array}$$

where $B=\mathrm{WA}=[b\left(\theta_{1}\right),\cdots ,b\left(\theta_{K}\right)]$ and $n_{b}\left(t\right)=\mathrm{Wn}\left(t\right)$ are the beamspace array manifold and noise, respectively. From Equation (5), the covariance matrix in beamspace is

(6) $$\begin{array}{cc} R & =E\left\{Y\left(t\right)Y^{H}\left(t\right)\right\}\\ & =BR_{s}B^{H}+V \end{array}$$

with $V=\sigma^{2}WW^{H}$. Inspired from [21], the covariance matrix in (5) can be represented in the Multiple Measurement Vectors (MMV) model as

(7) $$\begin{array}{cc} R & =BH+V\\ & =\sum_{k=1}^{K}c_{k}b\left(\theta_{k}\right)h_{k}+V \end{array}$$

where $H=R_{s}B^{H}$, $c_{k}h_{k}$ is the kth row vector of H and ${∥h_{k}∥}_{2}=1$.

### 2.2. Atomic Norm in Beamspace

As shown in [12], the element-space signals of the ULA in Equation (1) are consisted of distinct atoms over the atomic set

(8) $$A=\left\{{\left[a\left(\theta_{k}\right)\right]}_{n}=e^{-j\frac{2\pi nd\sin\theta }{\lambda }}|\theta_{k}\in \Xi \right\},$$

which can be represented by the atomic decomposition as

(9) $$x=\sum_{k=1}^{K}s_{k}a\left(\theta_{k}\right),\;a\left(\theta \right)\in A.$$

The atomic decomposition with the smallest total variation over A yields the $\ell_{1}$ atomic norm as

(10) $$\begin{array}{c} {∥x∥}_{A}=\inf\left\{\sum_{k=1}^{K}|s_{k}||x=\sum_{k=1}^{K}s_{k}a\left(\theta_{k}\right),a\left(\theta \right)\in A\right\}. \end{array}$$

Followed by generalized line spectral estimation framework in [17], the beamspace signal in Equation (5) can be retrieved by the following atomic norm minimization (ANM) problem:

(11) $$\begin{array}{c} \min_{x}{∥x∥}_{A}\\ s.t.\;y=\mathrm{Wx}, \end{array}$$

if the locations of sources are separated sufficiently. Followed by the Vandermonde decomposition theorem, the SDP implementation of Equation (11) can written as [12]

(12) $$\begin{array}{c} {∥x∥}_{T}=\min_{Q,w,z}\;\frac{1}{2N}\mathrm{Tr}\left(S\left(Q\right)\right)+\frac{1}{2}w\\ s.t.\;\left[\begin{array}{cc} S(Q) & x\\ x^{H} & w \end{array}\right]\geq 0,\\ \;\;\;y=\mathrm{Wx} \end{array}$$

where ${∥x∥}_{T}={∥x∥}_{A}$ and S(Q) is a Toeplitz matrix defined by Q.

It is noted that the method in Equation (11) can be applied to arbitrary beamspace design; however, it may result in high computational burden when the number of sensors is large, due to the fact that the computational complexity of SDP based implementation to Equation (12) depends on the dimension of x [22].

To develop the low complexity super resolution approach for the signal in beamspace, in this paper, we focus on the beamspace atomic norm

(13) $$\begin{array}{c} {∥y∥}_{B}=\inf\left\{\sum_{k=1}^{K}|{s^{\prime }}_{k}||y=\sum_{k=1}^{K}{s^{\prime }}_{k}b^{\prime }\left(\theta_{k}\right),b^{\prime }\left(\theta \right)\in B\right\} \end{array}$$

directly, with

(14) $$B=\left\{b^{\prime }\left(\theta \right)|b^{\prime }\left(\theta \right)=\mathrm{Wa}\left(\theta \right)/{∥\mathrm{Wa}(\theta )∥}_{F},\theta_{k}\in \Xi \right\}.$$

We will introduce an approximate low dimensional SDP implementation of the beamspace atomic norm minimization (BS-ANM) problem in Equation (13) for DFT beamspace design in the next section.

## 3. Low Dimensional Sdp Implementation for Bs-Anm

The generalized line spectral estimation based methods rely on the Vandermonde decomposition theorem. However, the beamspace array manifolds are not Vandermonde, which implies the major difficulty of realizing that the BS-ANM approach is to formulate the solvable convex optimization problem without the Vandermonde decomposition theorem. In this section, we propose an approximate low dimensional SDP implementation to the BS-ANM problem based on the super resolution theory on the semi-algebraic set [23].

We first introduce the dual problem of Equation (13) with Lagrangian analysis,

(15) maxΩReTryHΩs.t.ΩB*≤1,

which can retrieve the atomic decomposition of the ground truth if there exists the polynomial $q\left(\theta \right)=b^{\prime }H\left(\theta \right)\Omega$ satisfying the following dual certificate:

(16) $$\left\{\begin{array}{c} q(\theta_{k})=1,\forall k=1,2,\ldots ,K\\ {∥q(\theta )∥}_{2}<1,\forall \theta \neq \theta_{k} \end{array}\right.$$

where ${∥•∥}_{B}^{*}$ is the dual norm corresponding to ${∥•∥}_{B}$ that

(17) ΩB*=supGB≤1ReTrGHΩ=supθ∈Ξ,ρ2=1ReTrΩHb′(θ)ρ=supθ∈Ξ,ρ2=1TrρΩHb′(θ)=supθ∈ΞΩHb′(θ)2.

Letting $z=e^{-j\frac{2\pi nd\sin\theta }{\lambda }}$, we have

(18) $$\begin{array}{cc} \; & {∥\Omega ∥}_{B}^{*}\leq 1\\ \Leftrightarrow & F\left(z\right)=1-{b^{\prime }}^{H}\left(\theta \right)\Omega^{H}\Omega b^{\prime }\left(\theta \right)\geq 0, \end{array}$$

i.e., F(z) is a nonnegative trigonometric polynomial. In addition, F(z) is also a polynomial of b, which can be denoted as f(b), where b denotes b(θ) in Equation (5).

To insight the property of the polynomial f(b), we introduce the following theorems:

**Theorem** **1.** *Given a bounded semi-algebraic set*

(19) $$D\left(g\right)=\left\{\left.b\in R^{M}\right|g_{v}\left(b\right)\geq 0,v=1,2,\cdots ,V\right\},$$

*if there exists the nonnegative polynomial f(b)≥0 for any b∈D(g), we have*

(20) $$\begin{array}{c} f\left(b\right)=\sum_{\alpha \in {\left\{0,1\right\}}^{V}}g_{1}^{\alpha_{1}}\cdots g_{V}^{\alpha_{V}}s_{\alpha }, \end{array}$$

*with $g_{v}\left(b\right)\in R\left[b\right]$ and $s_{\alpha }\in \sum R{\left[b\right]}^{2}$, where R[b] and $\sum R{\left[b\right]}^{2}$ denote the set of real polynomials and real sum of squares polynomials on b, respectively.*

**Proof** **of** **Theorem** **1.** Theorem 1 can be found in [24] as Theorem 4.1 (See also Corollary 3 in [25]), thus we omit the proof here.  □

**Theorem** **2.** *The beamspace array manifold B in Equation (14) with the DFT beamspace in Equation (4) is a semi-algebraic set as D(g) in Equation (19).*

**Proof** **of** **Theorem** **2.** Followed by the invariant relationship between $b_{m}$ and $b_{m+1}$ in [4] and the property of trigonometric functions, where $b_{m}$ denotes the mth element of b(θ), we have

(21) $$\left\{\begin{array}{c} \begin{array}{c} v_{m}\left(b_{m},b_{m+1}\right)\sin\left(\frac{u}{2}\right)=p_{m}\left(b_{m},b_{m+1}\right)\cos\left(\frac{u}{2}\right),\\ \sin^{2}\left(\frac{u}{2}\right)+\cos^{2}\left(\frac{u}{2}\right)=1,\\ \sin\left(u\right)=2sin\left(\frac{u}{2}\right)\cos\left(\frac{u}{2}\right),\\ \cos\left(u\right)=2\cos^{2}\left(\frac{u}{2}\right)-1 \end{array} \end{array}\right.$$

with $u=\frac{2\pi nd}{\lambda }\sin\theta$ and

(22) $$\begin{array}{cc} v_{m}\left(b_{m},b_{m+1}\right) & =\cos\left(\frac{m\pi }{N}\right)b_{m}+\cos\left(\frac{(m+1)\pi }{N}\right)b_{m+1},\\ p_{m}\left(b_{m},b_{m+1}\right) & =\sin\left(\frac{m\pi }{N}\right)b_{m}+\sin\left(\frac{(m+1)\pi }{N}\right)b_{m+1}. \end{array}$$

Solving the polynomial system in Equation (21) implies that cos(u) and sin(u) can be determined by the polynomial of $\left\{b_{m},b_{m+1}\right\}$ uniquely, i.e., $\cos\left(u\right)=L_{m}\left(b_{m},b_{m+1}\right)$ and $\sin\left(u\right)=J_{m}\left(b_{m},b_{m+1}\right)$. Accordingly, we can represent $b_{m}$ as a polynomial of b such that $b_{m}=H_{m}\left(b\right)$.

Combining the constraints mentioned above together

(23) $$\left\{\begin{array}{c} L_{1}\left(b_{1},b_{2}\right)=L_{2}\left(b_{2},b_{3}\right)=\cdots =L_{M-1}\left(b_{M-1},b_{M}\right),\\ J_{1}\left(b_{1},b_{2}\right)=J_{2}\left(b_{2},b_{3}\right)=\cdots =J_{M}\left(b_{M-1},b_{M}\right),\\ L_{m}^{2}\left(b_{m},b_{m+1}\right)+J_{m}^{2}\left(b_{m},b_{m+1}\right)=1,\\ H_{m}\left(b\right)=b_{m},m=1,\cdots ,M, \end{array}\right.$$

it implies the solution of the polynomials system Equation (23) is the element of the beamspace atomic set B, i.e., the beamspace array manifold B is a semi-algebraic set as D(g), where $g_{v}\left(b\right)$ is constructed from Equation (23) and $g_{v}\left(b\right)\equiv 0$ for b∈B.  □

By utilizing Theorem 1 and Theorem 2, we present the following theorem.

**Theorem** **3.** *Given f(b) to be a polynomial of b, if f(b)≥0 on the domain B with the DFT beamspace in Equation (4), f(b) is a sum-of-squares polynomial on b.*

**Proof** **of** **Theorem** **3.** Given a beamspace steering vector b∈B, we have $\left|b_{m}\right|\leq 1$ followed by [4], which implies

(24) $$\chi \left(b\right)=M-\sum_{m=0}^{M}b_{m}^{2}\geq 0,b\in B,$$

and thus D(g) is bounded, where D(g) is the semi-algebraic set as D(g) in Equation (19) corresponding to B, followed by Theorem 2.

Using Theorem 1 and the fact that $g_{v}\left(b\right)\equiv 0$ for b∈B, we can say that any f(b)≥0 for b∈D(g) satisfies

(25) $$\begin{array}{cc} f(b) & =\sum_{\alpha \in {\left\{0,1\right\}}^{V}}g_{1}^{\alpha_{1}}\cdots g_{V}^{\alpha_{V}}s_{\alpha },\\ & =s_{0},s_{\alpha }\in \sum R{\left[b\right]}^{2}, \end{array}$$

where $s_{0}$ denotes the $s_{\alpha }$ with ${\left\{\alpha_{v}\right\}}_{v=1,\cdots ,V}=0$, i.e., f(b) is sum-of-squares polynomial on b.  □

We note that the result in Equation (25) is with the sum-of-squares relaxation of the polynomial in [26]. If $\deg\left(f\left(b\right)\right)=\deg\left(s_{0}\right)$, we can have the SDP implementation to Equation (15) followed by [24] as

(26) minP,Ω−ReTryHΩs.t.Tr(ΘnWHPW)=Tr(ΘnWHW)n∈HP−ΩH−ΩI≥0

where $\Theta_{n}\in R^{N\times N}$ is the zeros matrix except ones on the *n*th diagonal, *H* is a half space of [−(N−1),N−1]. The derivation of Equation (26) can be found in Appendix A.

Therefore, Equation (13) can be implemented by the following SDP problem with the standard Lagrangian analysis if strong duality holds

(27) $$\begin{array}{c} \min_{Q,M}\;\frac{1}{2}\mathrm{Tr}\left(WS\left(Q\right)W^{H}\right)+\frac{1}{2}\mathrm{Tr}\left(M\right)\\ s.t.\;\left[\begin{array}{cc} WS\left(Q\right)W^{H} & y\\ y^{H} & M \end{array}\right]\geq 0, \end{array}$$

where S(Q) is a Hermitian Toeplitz matrix with the first row vector Q. The derivation of Equation (27) can be found in Appendix B.

Regarding the Theorem 3 and its resulting SDP implementations of BS-ANM problem in Equations (26)–(27), we remark as follows:

**Remark** **1.** *It is well known that the positive trigonometric polynomial is sum-of-squares [24], i.e., $f\left(b\right)\in \sum R{\left[z\right]}^{2}$, which formulates the mathematical foundation of conventional ANM approaches, such as [12,17]. In Theorem 3, we further reveal that the positive polynomial $f\left(b\right)\in \sum R{\left[b\right]}^{2}$ for the DFT beamspace manifold. Based on this theorem, we develop the lower dimensional SDP implementation in Equations (26) and (27), which is our main contribution.*

**Remark** **2.** *Compared with the conventional one in Equation (12), the proposed SDP implementation in Equation (27) is with lower dimensional SDP constraints in the beamspace processing scenarios (N>M), which results in the low computational complexity as shown in Section 5.*

**Remark** **3.** *The SDP formulation in Equation (26) is based on the sum-of-squares relaxation [26], and thus is an approximate algorithm of Equation (15). It can be regarded as the optimal polynomial designing to regression the dual polynomial in Equation (16) under the polynomial order constraint as shown in [27].*

**Remark** **4.** *Our work is related with the ANM approaches on the semi-algebraic set [22,23]. In particular, we formulate the beamspace array manifold as the boundary of a semi-algebraic set, which is obviously a semi-algebraic set either, and this results in the low dimensional SDP implementation.*

## 4. BS-ANM via ADMM

Due to the SDP based methods with the high computational burden, we propose a fast implementation of BS-ANM approach via ADMM.

Let Λ≥0 and V≥0 be the Lagrangian multipliers of the constraint of Equation (27), the Lagrangian function of Equation (27) is

(28) $$\begin{array}{c} L\left(Q,M,\Lambda \right)=\mathrm{Tr}\left(M\right)+\langle V-\underset{T}{\underset{︸}{\left[\begin{array}{cc} WS\left(Q\right)W^{H} & y\\ y^{H} & M \end{array}\right]}},\Lambda \rangle \\ +\frac{\rho }{2}{∥V-\left[\begin{array}{cc} WS\left(Q\right)W^{H} & y\\ y^{H} & M \end{array}\right]∥}_{F}^{2}+\mathrm{Tr}\left(WS\left(Q\right)W^{H}\right) \end{array}$$

where $\Lambda =\left[\begin{array}{cc} \Lambda_{0} & \Omega \\ \Omega^{H} & \Lambda_{1} \end{array}\right]$, $V=\left[\begin{array}{cc} V_{0} & \Gamma \\ \Gamma^{H} & V_{1} \end{array}\right]$ and the blocks in Λ and V match the partition of the blocks in T. Then, the partial derivatives of L(Q,M,Λ) with respect to Q and M are calculated as follows:

(29) $$\begin{array}{c} \frac{\partial L}{\partial M}=I-\Lambda_{1}-\rho \left(V_{1}-M\right),\\ \frac{\partial L}{\partial Q}=e_{1}-E\left(\Lambda_{0},W\right)-\frac{\rho }{2}E\left(V_{0},W\right)+\frac{\rho }{2}F\left(W\right)\left[\begin{array}{c} Q_{r}\\ Q_{i} \end{array}\right], \end{array}$$

where $Q_{r}$ and $Q_{i}$ denote the real and imaginary part of Q, $e_{1}$ is the vector with zeros except the first element, I is the unity matrix. $E\left(V_{0},W\right)\in C^{N\times 1}$ is a vector function of $V_{0}$ and W, whose nth element is ${\left[E(V_{0},W)\right]}_{n}=\mathrm{Tr}\left(\Theta_{n}W^{H}V_{0}W\right)$ and $E(\Lambda_{0},W)$ is defined similarly. $F\left(W\right)=\left[F_{r}\left(W\right)\;-jF_{i}\left(W\right)\right]$ is a matrix function of W, where the element of $F_{r}\left(W\right)\in C^{N\times N}$ on mth row and nth column can be described as

(30) $$\begin{array}{c} {\left[F_{r}\left(W\right)\right]}_{m,n}=\mathrm{Tr}\left(W\Theta_{n}W^{H}\Pi_{m}\right)\\ {\left[F_{i}\left(W\right)\right]}_{m,n}=\mathrm{Tr}\left(W\Theta_{n}W^{H}\Upsilon_{m}\right) \end{array}$$

where

(31) $$\begin{array}{cc} \Pi_{m} & =\left\{\begin{array}{c} W\Theta_{m}W^{H}+W\Theta_{-m}W^{H},m\neq 0\\ WW^{H},m=0, \end{array}\right.\\ \Upsilon_{m} & =\left\{\begin{array}{c} -W\Theta_{m}W^{H}+W\Theta_{-m}W^{H},m\neq 0\\ WW^{H},m=0. \end{array}\right. \end{array}$$

Therefore, we can give the updates steps for the alternating optimization of the ADMM. Given the results of lth iteration $Q^{l},\Lambda_{0}^{l},\Lambda_{1}^{l},V_{1}^{l},V_{0}^{l}$, the updates rules can be described as

(32) $$\begin{array}{cc} M^{l+1} & =\frac{1}{\rho }\Lambda_{1}^{l}+V_{1}^{l}-\frac{1}{\rho }I \end{array}$$

(33) $$\begin{array}{cc} \left[\begin{array}{c} Q_{r}^{l+1}\\ Q_{i}^{l+1} \end{array}\right] & =\frac{2}{\rho }F^{†}\left(W\right)\left[E\left(\Lambda_{0}^{l},W\right)+\frac{\rho }{2}E\left(V_{0}^{l},W\right)-e_{1}\right] \end{array}$$

(34) $$\begin{array}{cc} \Lambda^{l+1} & =\Lambda^{l}+\rho \left(V^{l+1}-T^{l+1}\right) \end{array}$$

with

(35) $$\begin{array}{cc} T^{l+1} & =\left[\begin{array}{cc} WS\left(Q^{l+1}\right)W^{H} & y\\ y^{H} & M^{l+1} \end{array}\right] \end{array}$$

(36) $$\begin{array}{cc} V^{l+1} & ={\left[T^{l+1}-\rho \Lambda^{l}\right]}_{+}. \end{array}$$

In Equation (33), ${\left[•\right]}_{+}$ denotes the orthogonal projection on the positive semidefinite matrices, which is defined as

(37) $$\begin{array}{cc} V^{l+1} & =U^{l}\Sigma_{+}{U^{l}}^{H} \end{array}$$

where $T^{l+1}-\rho \Lambda^{l}=U^{l}\Sigma {U^{l}}^{H}$ is the eigenvalue decomposition and $\Sigma_{+}$ is Σ with all negative eigenvalue being zeros.

Summarizing above, we completed the iterations steps of ADMM algorithm for the BS-ANM problem in Table 1. The iteration will stop until converging or achieving the iteration number limits.

## 5. Complexity Analysis

In this section, we provide the complexity analysis to the implementations of the ANM based methods in beamspace, such as the algorithms in Equations (12) and (27), Table 1 and [28].

### 5.1. Complexity of SDP Implementations

It is shown in [22] that a primal-dual path following method needs C(ϵ)=−O(1)ln(ϵ)S flops to solve the SDP problem with accuracy ϵ, where

(38) $$S={\left\{1+\sum_{i=1}^{I}\beta_{i}\right\}}^{\frac{1}{2}}\left\{\gamma^{3}+\gamma^{2}\sum_{i=1}^{I}\beta_{i}^{2}+\gamma \sum_{i=1}^{I}\beta_{i}^{3}\right\}$$

and the SDP problem is with γ independent real variables and *I* real linear matrix inequalities (LMI) with the ith LMI size of β×β.

Firstly, we analyze the GL based SDP implementation in Equation (12). Here, we have γ=4N−2M,I=1 and $\beta_{1}=2\left(N+1\right)$, and thus $S=O(N^{4.5})$ for Equation (12), followed by [22].

For the SDP implementation of BS-ANM approach in Equation (27), there are N−1 complex variables, two real variables, and a (M+1)×(M+1) SDP constraint, thus we have γ=2N,I=1 and $\beta_{1}=2\left(M+1\right)$, which gives

(39) $$S=\sqrt{2M+3}\left\{8N^{3}+16N^{2}\left(M+1\right)+16N{\left(M+1\right)}^{3}\right\}.$$

Accordingly, for N>M, the computational complexity of Equation (27) is $O(NM^{3.5})$, compared with the computational complexity of $S=O(N^{4.5})$ for the GL based method.

### 5.2. Complexity of ADMM Based Methods

The ADMM based method for generalized line spectral estimation problem has been proposed in [28]. As shown in [28], the step with highest computational burden of the ADMM methods is the projection onto the semidefinite cone as Equation (34), and thus the ADMM based method for GL problem is with the complexity of $O(N^{3})$, since the eigenvalue decomposition requires $O(N^{3})$ operations.

Similarly, the algorithm proposed in Section 4 is with a semidefinite cone projection operator of M×M matrix, and the complexity of the proposed ADMM method is $O(M^{3})$ accordingly. It is noted that the complexity of the MUSIC [29] is $O(M^{3})$, although the proposed ADMM method has a higher computational cost than the MUSCI method since the iterations.

Therefore, the proposed methods are significantly more computationally efficient than GL based ones, when N>M. The complexity gain of the proposed methods is $O{\left(\frac{M}{N}\right)}^{3.5}$ for SDP implementation and $O{\left(\frac{M}{N}\right)}^{3}$ for ADMM implementation, respectively.

## 6. DOA Estimation via BS-ANM

Based on the BS-ANM approaches proposed in Section 3 and Section 4, we develop the covariance matrix based DOA estimation algorithm in free space and the channel estimation method for massive MIMO system with lens antennas.

### 6.1. DOA Estimation with Covariance Matrix

Rewriting the MMV model in Equation (7) that $R=\sum_{k=1}^{K}c_{k}b\left(\theta_{k}\right)h_{k}$, we define the beamspace atomic set as

(40) $$B^{\prime }:=\left\{b^{\prime }\left(\theta \right)h\left|b^{\prime }\left(\theta \right)=\mathrm{Wa}\left(\theta \right)/{∥\mathrm{Wa}(\theta )∥}_{F},\theta \in \Xi ,{∥h∥}_{2}=1\right.\right\}.$$

Then, the beamspace atomic $\ell_{1}$ norm of the covariance matrix R can be defined as

(41) $$\begin{array}{c} {∥R∥}_{B}^{\prime }=\inf\left\{\left.\sum_{k=1}^{K}c_{k}\right|R=\sum_{k=1}^{K}c_{k}b^{\prime }\left(\theta_{k}\right)h_{k}.\right\}. \end{array}$$

Followed by Equation (27), the SDP implementation of Equation (41) can be written as

(42) $$\begin{array}{cc} \; & \min_{Q,M}\;\frac{1}{2}\mathrm{Tr}\left(WS\left(Q\right)W^{H}\right)+\frac{1}{2}\mathrm{Tr}\left(M\right)\\ \; & s.t.\;\left[\begin{array}{cc} WS\left(Q\right)W^{H} & R\\ R^{H} & M \end{array}\right]\geq 0. \end{array}$$

In practice DOA estimation applications, we usually estimate the noisy covariance matrix as

(43) $$\begin{array}{cc} \hat{R} & =\frac{1}{J}\sum_{t=1}^{J}Y\left(t\right)Y^{H}\left(t\right),\\ \; & =BR_{s}B^{H}+\eta , \end{array}$$

where η is the outlier term. Consequently, the BS-ANM approach can be applied to the noisy case via regularization:

(44) $$\begin{array}{cc} \; & \min_{Q,M,R}\;\frac{1}{2}\mathrm{Tr}\left(WS\left(Q\right)W^{H}\right)+\frac{1}{2}\mathrm{Tr}\left(M\right)\\ \; & s.t.\;\left[\begin{array}{cc} WS\left(Q\right)W^{H} & R\\ R^{H} & M \end{array}\right]\geq 0,\\ \; & \;\;\;{∥\hat{R}-R∥}_{2}^{2}\leq \varepsilon^{2} \end{array}$$

where ε can be set as $\varepsilon =M\sigma^{2}$, followed by [30]. Here, we estimate σ with the square root of the smallest eigenvalue of $\hat{R}$. Then, the DOAs can be obtained by employing Root-MUSIC [31] to $WS\left(Q^{☆}\right)W^{H}$, where $Q^{☆}$ is the solution of Equation (44).

Summarizing above, we list the steps of the DOA estimation algorithm via the BS-ANM approach in Table 2.

To apply the ADMM approach to DOA estimation, we propose a subspace based method, inspired from [32]. Consider the covariance matrix in Equation (43)

(45) $$\hat{R}=U_{s}\Sigma_{s}U_{s}^{H}+\sigma^{2}U_{n}U_{n}^{H},$$

where $U_{s}$ and $U_{n}$ are the signal and noise subspace, respectively, which is obtained by eigenvalue decomposition. Let $\tilde{y}=U_{s}U_{s}^{†}$ be substituted into Equations (32), (33), and (34) to replace y; the proposed ADMM method can be applied to DOA estimation in noisy environments. We list the algorithm in Table 3.

### 6.2. Channel Estimation with Lens Antenna Array

In the millimeter-wave massive MIMO system, the lens antenna arrays with the DFT beamformer are used for the hardware cost and power reduction [9,10]. In this subsection, we apply the BS-ANM approach to the channel estimation at the base station (BS) in such scenario.

Considering a narrowband millimeter-wave massive MIMO system with *N* antennas ULA at base station and a single antenna mobile phone, the uplink channel can be expressed as [11]

(46) $$H\left(t\right)=\sum_{k=1}^{K}g_{k}\left(t\right)a\left(\theta_{k}\right),$$

where a(θ) is the steering vector of ULA at BS in Equation (3) and $g_{k}\left(t\right)$ denotes the channel gain for the kth propagation path at time block *t*.

As shown in [9], given a lens antenna array with the *M* channel DFT beamformer, the received signal after normalizing the known pilot sequence can be given by

(47) $$\begin{array}{cc} y(t) & =Bg\left(t\right)+n_{b}\left(t\right) \end{array}$$

where $g\left(t\right)={\left[g_{1}\left(t\right),\cdots ,g_{K}\left(t\right)\right]}^{T}$, B is the beamspace array manifold in Equation (5), then the channel estimation can be regarded as the sparse representation problem with the MMV model.

Thus, the estimated angle of the scatters $\left\{{\hat{\theta }}_{k}\right\}$ can be obtained by the algorithm presented in Section 6.1 and the path gain $\left\{g_{k}\left(t\right)\right\}$ can be given by

(48) $$\begin{array}{cc} \; & \hat{g}\left(t\right)=\min_{g}\;{∥y(t)-\mathrm{Bg}(t)∥}_{2} \end{array}$$

Finally, we have the estimated channel state information (CSI) as

(49) $$\hat{H}\left(t\right)=\sum_{k=1}^{K}{\hat{g}}_{k}\left(t\right)a\left({\hat{\theta }}_{k}\right).$$

The steps of the BS-ANM based channel estimation algorithm for the lens antenna array are presented in Table 4.

## 7. Simulations

In this section, we evaluate the performance of the proposed methods through simulations. Here, the proposed SDP and ADMM based methods are referred to as BS-ANM and BS-ADMM, respectively.

**Figure 1:**
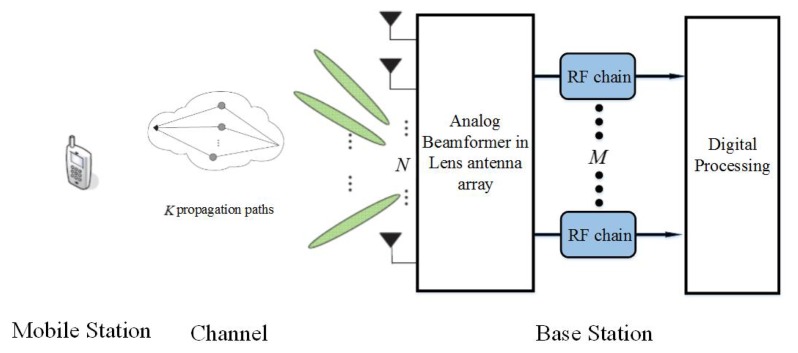
The architecture of the massive MIMO system with lens antenna array.

Firstly, we compare the resolution capability of the BS-ANM and the generalized line spectral estimation based ANM method (GL-ANM). Consequently, we demonstrate the computational complexity and the DOA estimation performance comparison of the proposed the BS-ANM and BS-ADMM methods with the conventional ANM and subspace based methods, such as Beamspace MUSIC in [29], SPA in [13], and GL-ANM in [17]. As a performance metric of DOA estimation accuracy, the Cramer–Rao bound (CRB) derived in [33] is also presented in simulations. In Section 7.1, Section 7.2 and Section 7.3, simulations perform the DOA estimation in free space, where the signal model is followed from Equation (5). In Section 7.4, we show the performance of the proposed methods for the channel estimation problem in the massive MIMO system as shown in Figure 1.

In simulations of DOA estimation, the root mean square error (RMSE) is utilized to evaluate the performance of algorithms as

(50) $$\mathrm{RMSE}=\sqrt{\frac{1}{TK}\sum_{i=1}^{K}\sum_{j=1}^{T}{\left({\overset{⌢}{\theta }}_{ij}-\theta_{i}\right)}^{2}}$$

where ${\overset{⌢}{\theta }}_{ij}$ and $\theta_{i}$ denote the estimated and the true DOA of the *i*th target at *j*th trial, the number of trials is *T* and *K* is the number of sources. Monte Carlo simulations are are performed with T=200 to compute RMSE using Intel i7-8700K CPU PC (Santa Clara, CA, USA). All the simulations are implemented by Matlab2017b (Natick, MA, USA). Our code is publicly available at *github.com/panda-1982/BS-ANM*.

### 7.1. Comparison of Resolution and Complexity

**Figure 2:**
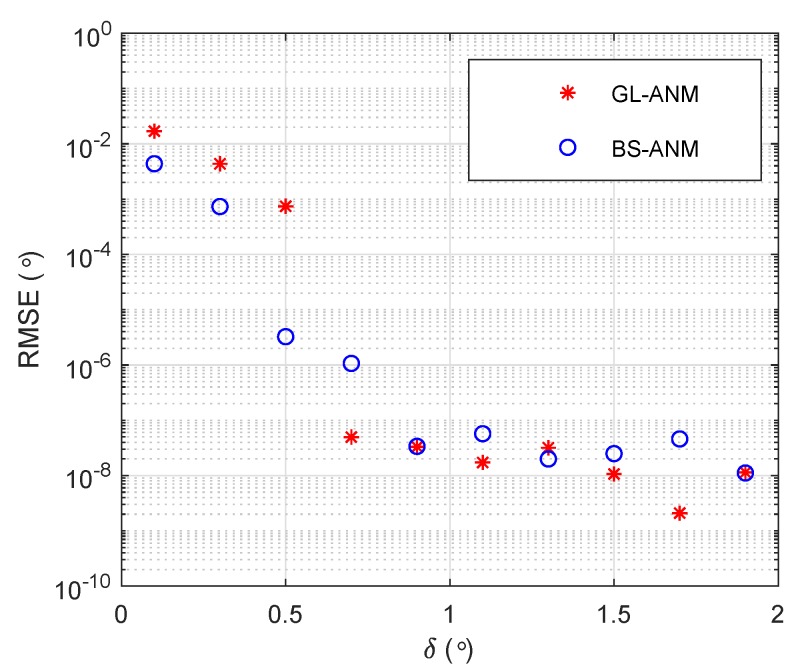
RMSE versus angle separation δ for the GL-ANM and BS-ANM methods.

Consider a ULA with N=22 elements and M=10 DFT beamformer, d=λ/2, there are two sources at $\theta ={-20}^{\circ }$ and $\theta ={-20+\delta }^{\circ }$. The RMSE of the BS-ANM method and GL-ANM method in a noiseless case with 100 snapshots are shown in Figure 2 versus angle separation. It can be seen that the BS-ANM method performs similar resolution capacity with the GL-ANM method.

**Figure 3:**
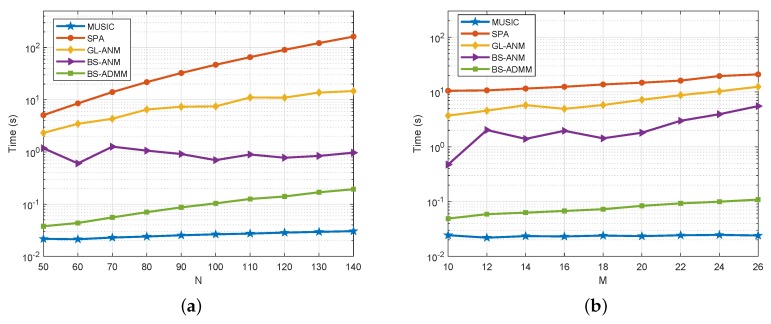
(**a**) Runtime versus the number of sensors *N*; (**b**) Runtime versus the dimension of the beamspace *M*.

The computing time of GL-ANM, BS-ANM, BS-ADMM, SPA, and MUSIC methods are presented in Figure 3. In Figure 3a, the dimension of beamspace is fixed as M=10, and we compare the CPU runtime of evaluated methods versus the number of sensors *N*. In Figure 3b, the number of sensors is set as N=64 and the CPU runtime of evaluated methods is compared versus the dimension of beamspace *M*. In simulations, the GL-ANM, BS-ANM, and SPA methods are implemented by the CVX software package [34], and the BS-ADMM is implemented in Matlab with the "codegen" feature. As shown from the results, the runtime of GL-ANM is significantly higher than BS-ANM and BS-ADMM, especially when the number of sensors *N* is large.

### 7.2. Performance for Uncorrelated Sources

In this subsection, the proposed BS-ANM based methods are evaluated with some baselines in the presence of uncorrelated sources.

**Figure 4:**
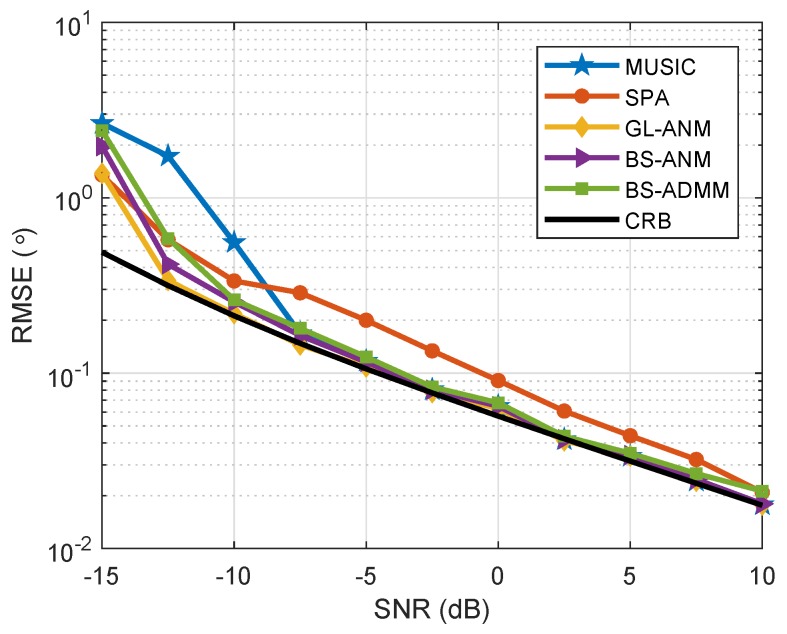
RMSE comparison for uncorrelated sources at $\theta =5^{\circ }$ and θ = −10°, J=200 when SNR varies from −15 to 10 dB.

Supposing that the array configuration is the same as the setting in Section 7.1, there are two independent sources at $\theta ={-10}^{\circ }$ and $\theta =5^{\circ }$ impinging onto the array and 200 snapshots are collected in each trial. Figure 4 shows the RMSE of DOA estimation of the evaluated algorithms when SNR varies from −15 to 10 dB. It can be seen that the proposed BS-ANM and BS-ADMM methods have almost the identical RMSE to GL-ANM method and perform better than the other competitors. We also note that the performance of MUSIC method degrades significantly compared with the sparsity based methods when SNR is lower than −8 dB.

**Figure 5:**
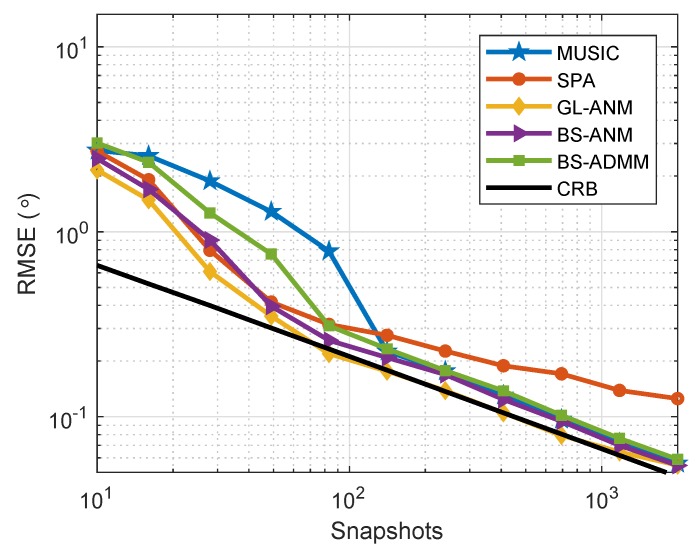
RMSE comparison for uncorrelated sources at $\theta =5^{\circ }$ and θ = −10°, SNR = −10 dB when the number of snapshots varies from 10 to 2000.

When the SNR is fixed at SNR = −10 dB, Figure 5 plots the RMSE of DOA estimation of the evaluated algorithms versus a different number of snapshots. The result shows that the GL-ANM method performs the best among the algorithms, and the BS-ANM method has a similar performance to the GL-ANM method. When the number of snapshot is not too low, i.e., larger than 100, the BS-ADMM method also shows the satisfying DOA estimation accuracy. In addition, it is shown that the MUSIC method suffers from the lack of snapshots more seriously than the sparsity based methods.

**Figure 6:**
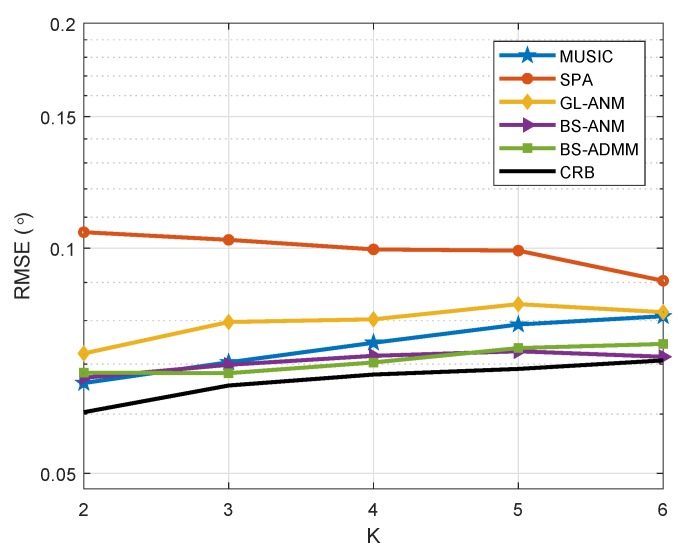
RMSE comparison for uncorrelated sources at $\left[{-30}^{\circ },{-24}^{\circ },\cdots ,{\left(6K-36\right)}^{\circ }\right]$, J=100 and SNR = 0 dB when the number of sources varies from 2 to 6.

In Figure 6, we demonstrate the DOA estimation performance versus the number of sources. Considering the ULA with N=22 elements and M=8 DFT beamformer, there are *K* uncorrelated sources at $\left[{-30}^{\circ },{-24}^{\circ },\cdots ,{\left(6K-36\right)}^{\circ }\right]$ and J=100 snapshots are used for DOA estimation. The SNR is fixed at 0dB. We can see from the simulation results that the proposed BS-ANM and BS-ADMM methods are robust to the multiple sources’ scenarios.

### 7.3. Performance for Correlated Sources

**Figure 7:**
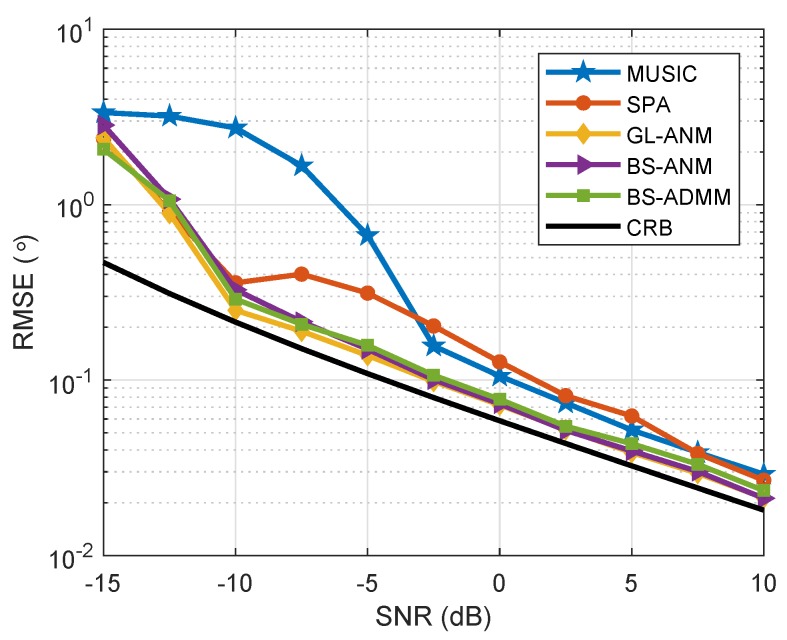
RMSE comparison for correlated sources at $\theta =5^{\circ }$ and $\theta ={-10}^{\circ }$, J=200 when SNR varies from −15 to 10 dB, ζ=0.5.

Next, we study the effect of correlated sources on the evaluated algorithms. Supposing that the array configuration and the source location are set as in Figure 4, the signal sequences consisted of 200 snapshots in each trial with the correlation coefficient of ζ=0.5. We present the RMSE of the evaluated algorithms versus SNR in Figure 7. As we can see from Figure 7, GL-ANM, BS-ANM, and BS-ADMM methods have almost the same performance, which is very close to CRB when SNR is higher than −10 dB. In addition, the SPA and MUSIC methods are suffered from the correlated sources more seriously than the ANM based ones, especially in low SNR.

**Figure 8:**
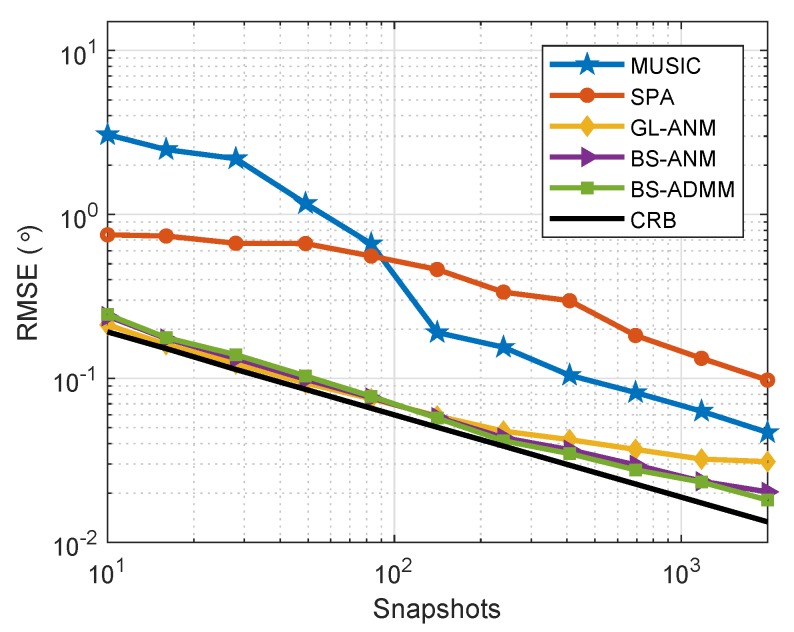
RMSE comparison for correlated sources at $\theta =5^{\circ }$ and $\theta ={-10}^{\circ }$, SNR = 0 dB when the number of snapshots varies from 10 to 2000, ζ=0.7.

To evaluate the effect of the number of snapshots in a correlated sources’ scenario, we set SNR=0dB and the correlation coefficient of ζ=0.7, and the RMSE of the evaluated algorithms versus the number of snapshots is demonstrated in Figure 8. The simulation results show that the proposed BS-ANM and BS-ADMM methods provide the significant robustness against the correlation of the sources compared with SPA and MUSIC methods and can achieve satisfying DOA estimation performance close to CRB even with a low number of snapshots.

### 7.4. Performance for Channel Estimation

We verify the channel estimation method proposed in Section 6.2 with lens antenna array for massive MIMO system as shown in Figure 1.

Suppose the N=64 ULA with M=16 dimensions DFT beamspace as the receiver antennas array and the single antenna transmitter consist of the MIMO system, there are K=6 random distributed scatters in the main-lobe region of the receiver array whose locations are satisfying uniform distribution, and the path gains $\left\{g_{k}\left(t\right)\right\}$ are assumed as independent identical Gaussian distribution. The channel estimation is carried out with J=10 snapshots and η=9 in each trail. The 200 Monte Carlo trials are performed in the simulation. The normalized mean squared error (NMSE) of the CSI estimation

(51) $$\mathrm{NMSE}=\frac{1}{L}\sum_{l=1}^{L}\frac{{∥{\hat{H}}_{l}\left(t\right)-H\left(t\right)∥}_{F}^{2}}{{∥H(t)∥}_{F}^{2}}$$

is utilized to evaluate the performance of the channel estimation methods, where ${\hat{H}}_{l}\left(t\right)$ and H(t) are the estimated and ground truth CSI, respectively.

**Figure 9:**
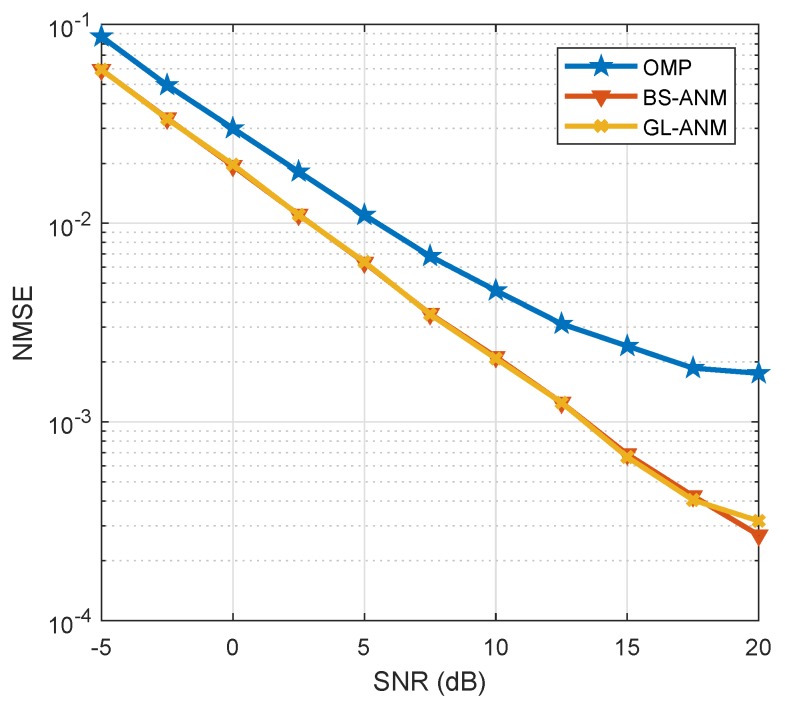
NMSE comparison of different channel estimation methods when SNR varies from −5 to 20 dB.

The NMSE of the orthogonal matching pursuit (OMP) based channel estimation method in [8], the GL based channel estimation method in [20] and the proposed method in Section 6.2 versus SNR is shown in Figure 9. The simulation results indicate that the proposed method shows the significant performance improvement over the OMP based method and has almost the same performance as the GL based method.

## 8. Conclusions

In this paper, we study the low complexity implementation of the beamspace atomic norm minimization for DOA estimation. We propose the beamspace atomic norm and its low dimensional SDP implementation for DFT beamspace. By utilizing this approach, the covariance based BS-ANM DOA estimation methods and BS-ANM based channel estimation method for a massive MIMO system with a lens antenna array are developed. The complexity analysis and simulations indicate that the proposed methods have almost the same performance and significantly lower computational complexity than the generalized line spectral estimation based ANM methods. In addition, the proposed methods demonstrate the performance improvement compared to some state-of-the-art counterparts.

## Figures and Tables

**Table 1:** The ADMM based BS-ANM algorithm.

Initialize: $Q^{0}=0,M^{0}=0,\Lambda^{0}=0,V^{0}=0,l=0$
Input: *y*; Output: $V_{o}$
**Iteration:**
**While** (the stop condition is not satisfied) **do**:
1: Update $M^{l+1}$ and $Q^{l+1}$ using Equations (32) and (33), respectively.
2: Update $T^{l+1}$ using Equation (35).
3: Update $V^{l+1}$ using Equation (36).
4: Update $\Lambda^{l+1}$ using Equation (34).
5: l=l+1;
**end**
**Output:** $V_{o}=V_{l}$

**Table 2:** DOA estimation algorithm via BS-ANM.

Inpute: Y(t),K
Output: ${\left\{{\hat{\theta }}_{k}\right\}}_{k=1,2,\cdots ,K}$
1: Compute the covariance matrix $\hat{R}$ as in Equation (43).
2: Compute the smallest of eigenvalue of $\hat{R}$ to obtain ε.
3: Solve Equation (44) to obtain $WS\left(Q^{☆}\right)W^{H}$.
4: Apply Root-MUSIC to $WS\left(Q^{☆}\right)W^{H}$ and estimate ${\left\{{\hat{\theta }}_{k}\right\}}_{k=1,2,\cdots ,K}$.

**Table 3:** The DOA estimation algorithm via ADMM.

Input: Y(t),K
Output: ${\left\{{\hat{\theta }}_{k}\right\}}_{k=1,2,\cdots ,K}$
1: Compute the covariance matrix $\hat{R}$ as in Equation (43).
2: Compute the eigenvalue decomposition of $\hat{R}$ and obtain $U_{s}$ as the eigenvectors corresponding to the large eigenvalues.
3: Let $y=U_{s}U_{s}^{†}$ and compute $V_{o}$ by the algorithm in Table 1.
4: Apply Root-MUSIC to $V_{o}$ to estimate ${\left\{{\hat{\theta }}_{k}\right\}}_{k=1,2,\cdots ,K}$.

**Table 4:** Channel estimation algorithm via BS-ANM.

Input: y(t)
Output: $\hat{H}\left(t\right)$
1: Compute the covariance matrix $\hat{R}$ with y(t) as in Equation (43).
2: Solve Equation (44) to obtain $WS\left(Q^{☆}\right)W^{H}$.
3: Compute the eigenvalue of $WS\left(Q^{☆}\right)W^{H}$ as $\gamma_{1}\geq \gamma_{2}\geq \cdots \geq \gamma_{M}$, and estimate the number of scatters by $K=\min[\eta ,\sup\left\{k:\gamma_{k}\geq 0.01\gamma_{1}\right\}]$.
4: Apply Root-MUSIC to $V_{o}$ to estimate ${\left\{{\hat{\theta }}_{k}\right\}}_{k=1,2,\cdots ,K}$.
5: Estimate the path gain $\hat{g}\left(t\right)$ using Equation (48).
6: Estimate $\hat{H}\left(t\right)$ by Equation (49).

## Appendix A. Derivation of Equation (26)

Followed by $s_{0}\in \sum R{\left[b\right]}^{2}$ and $\deg\left(f\left(b\right)\right)=\deg\left(s_{0}\right)$, we have a positive semidefinite matrix Σ≥0 that

(A1) $$\begin{array}{cc} {∥\Omega ∥}_{B}^{*}\leq 1 & \Leftrightarrow 1-b{{}^{\prime }}^{H}\left(\theta \right)\Omega \Omega^{H}b^{\prime }\left(\theta \right)\geq 0\\ \; & \Leftrightarrow b^{H}\left(\theta \right)b\left(\theta \right)-b^{H}\left(\theta \right)\Omega^{H}\Omega b\left(\theta \right)\geq 0,\\ \; & \Leftrightarrow a^{H}\left(\theta \right)W^{H}\mathrm{Wa}\left(\theta \right)-a^{H}\left(\theta \right)W^{H}\Omega^{H}\Omega \mathrm{Wa}\left(\theta \right)=a^{H}\left(\theta \right)W^{H}\Sigma \mathrm{Wa}\left(\theta \right). \end{array}$$

Let $P=\Sigma +\Omega^{H}\Omega$, according to the Gram matrix representation of positive polynomials in [24], Equation (A1) gives

(A2) $$\begin{array}{cc} a^{H}\left(\theta \right)W^{H}\mathrm{PWa}\left(\theta \right) & =\mathrm{Tr}(a\left(\theta \right)a^{H}\left(\theta \right)W^{H}\mathrm{PW})\\ \; & =\sum_{n}\left[\Theta_{n}W^{H}P^{*}W\right]z^{n}\\ \; & =\sum_{n}\left[\Theta_{n}W^{H}W\right]z^{n}, \end{array}$$

and thus

(A3) $${∥\Omega ∥}_{B}^{*}\leq 1\Leftrightarrow \left\{\begin{array}{c} \mathrm{Tr}\left(\Theta_{n}W^{H}\mathrm{PW}\right)=\mathrm{Tr}\left(\Theta_{n}W^{H}W\right),n\in H,\\ \left[\begin{array}{cc} P & -\Omega^{H}\\ -\Omega & I \end{array}\right]\geq 0, \end{array}\right.$$

which implies the SDP implementation of Equation(15) as

(A4) minP,Ω−ReTryHΩs.t.Tr(ΘnWHPW)=Tr(ΘnWHW),n∈HP−ΩH−ΩI≥0

## Appendix B. Derivation of Equation (27)

Let $\Lambda =\left[\begin{array}{cc} S & Z\\ Z^{H} & M \end{array}\right]\geq 0$ and Q be the Lagrangian multipliers of the two constraints of Equation (27), the Lagrangian function of Equation (27) can be given as

(A5) L(Q,M,S,Z)=−ReTr(yΩH)+∑n∈Hqn(Tr(ΘnWHPW)−Tr(ΘnWHW))−TrP−Ω−ΩHIΛ=−12Tr(yΩH+ΩyH)−Tr(PS−ΩZH−ΩHZ+M)+∑n∈Hqn(Tr(ΘnWHPW)−Tr(ΘnWHW))

For minimizing L(Q,M,S,Z), we can derive the partial derivative of L(Q,M,S,Z) with respect to (P,Ω), which results in that

(A6) $$\left\{\begin{array}{cc} S & =W\left[\sum_{n\in H}q_{n}\Theta_{n}\right]W^{H}\\ \frac{1}{2}y & =Z \end{array}.\right.$$

Inserting Equation (A6) into Equation (A5), we have

(A7) $$\inf\left(L\right)=-\mathrm{Tr}(WS\left(Q\right)W^{H})-\mathrm{Tr}\left(M\right),$$

which results in the dual problem of Equation (26) as

(A8) $$\begin{array}{cc} \; & \min_{Q,M}\;\mathrm{Tr}\left(WS\left(Q\right)W^{H}\right)+\mathrm{Tr}\left(M\right)\\ \; & s.t.\;\left[\begin{array}{cc} WS\left(Q\right)W^{H} & \frac{1}{2}R\\ \frac{1}{2}R^{H} & M \end{array}\right]\geq 0, \end{array}$$

that is,

(A9) $$\begin{array}{cc} \; & \min_{Q,M}\;\frac{1}{2}\mathrm{Tr}\left(WS\left(Q\right)W^{H}\right)+\frac{1}{2}\mathrm{Tr}\left(M\right)\\ \; & s.t.\;\left[\begin{array}{cc} WS\left(Q\right)W^{H} & R\\ R^{H} & M \end{array}\right]\geq 0. \end{array}$$

## References

[1] Zhao H., Zhang L., Shen Y. On the Optimal Beamspace Design for Direct Localization Systems. Proceedings of the 2018 IEEE International Conference on Communications (ICC).

[2] Zhao H., Zhang N., Shen Y. Robust Beamspace Design for Direct Localization. Proceedings of the 2019 IEEE International Conference on Acoustics, Speech and Signal Processing (ICASSP).

[3] Wang X., Aboutanios E. Adaptive Reduced-Dimensional Beamspace Beamformer Design by Analogue Beam Selection. Proceedings of the 2019 IEEE International Conference on Acoustics, Speech and Signal Processing (ICASSP).

[4] Zoltowski M.D., Haardt M., Mathews C.P. (1996). Closed-form 2D angle estimation with rectangular arrays in element space or beamspace via unitary ESPRIT. IEEE Trans. Signal Process..

[5] Hassanien A., Vorobyov S.A. (2009). A Robust Adaptive Dimension Reduction Technique With Application to Array Processing. IEEE Signal Process. Lett..

[6] Tang B., Tang J., Zhang Y., Zheng Z. (2013). Maximum likelihood estimation of DOD and DOA for bistatic MIMO radar. Signal Process..

[7] Wen F., Mao C., Zhang G. (2019). Direction finding in MIMO radar with large antenna arrays and nonorthogonal waveforms. Digit. Signal Process..

[8] Lee J., Gil G.T., Lee Y.H. (2016). Channel estimation vai orthogonal matching pursuit for hybrid MIMO systems in millimeter wave communications. IEEE Trans. Commun..

[9] Gao X., Dai L., Han S., I C., Wang X. (2017). Reliable Beamspace Channel Estimation for Millimeter-Wave Massive MIMO Systems with Lens Antenna Array. IEEE Trans. Wirel. Commun..

[10] Cheng X., Yang Y., Xia B., Wei N., Li S. (2019). Sparse Channel Estimation for Millimeter Wave Massive MIMO Systems With Lens Antenna Array. IEEE Trans. Veh. Technol..

[11] Wang Y., Zhang Y., Tian Z., Leus G., Zhang G. (2019). Super-Resolution Channel Estimation for Arbitrary Arrays in Hybrid Millimeter-Wave Massive MIMO Systems. IEEE J. Sel. Top. Signal Process..

[12] Tang G., Bhaskar B.N., Shah P., Recht B. (2013). Compressed Sensing Off the Grid. IEEE Trans. Inf. Theory.

[13] Yang Z., Xie L., Zhang C. (2014). A Discretization-Free Sparse and Parametric Approach for Linear Array Signal Processing. IEEE Trans. Signal Process..

[14] Wu X., Zhu W., Yan J. (2017). A Toeplitz Covariance Matrix Reconstruction Approach for Direction-of-Arrival Estimation. IEEE Trans. Veh. Technol..

[15] Steffens C., Pesavento M., Pfetsch M.E. (2018). A Compact Formulation for the *ℓ*_2,1_ Mixed-Norm Minimization Problem. IEEE Trans. Signal Process..

[16] Zhou C., Gu Y., Fan X., Shi Z., Mao G., Zhang Y.D. (2018). Direction-of-Arrival Estimation for Coprime Array via Virtual Array Interpolation. IEEE Trans. Signal Process..

[17] Heckel R., Soltanolkotabi M. (2018). Generalized Line Spectral Estimation via Convex Optimization. IEEE Trans. Inf. Theory.

[18] Ardah K., de Almeida A.L.F., Haardt M. A Gridless CS Approach for Channel Estimation in Hybrid Massive MIMO Systems. Proceedings of the 2019 IEEE International Conference on Acoustics, Speech and Signal Processing (ICASSP).

[19] Chu H., Zheng L., Wang X. (2019). Super-Resolution mmWave Channel Estimation for Generalized Spatial Modulation Systems. IEEE J. Sel. Top. Signal Process..

[20] Chu H., Zheng L., Wang X. (2018). Semi-Blind Millimeter-Wave Channel Estimation Using Atomic Norm Minimization. IEEE Commun. Lett..

[21] Yin J., Chen T. (2011). Direction-of-Arrival Estimation Using a Sparse Representation of Array Covariance Vectors. IEEE Trans. Signal Process..

[22] Mahata K., Hyder M.M. (2018). Fast Frequency Estimation With Prior Information. IEEE Trans. Signal Process..

[23] De Castro Y., Gamboa F., Henrion D., Lasserre J.B. (2017). Exact solutions to super resoluion on semi-algebraic domains in high dimensions. IEEE Trans. Inf. Theroy.

[24] Dumitrescu B. (2007). Positive Trigonometric Polynomials and Signal Processing Applications.

[25] Schmudgen K. (1991). The K-moment problem for compact semi-algebraic sets. Math. Annalen.

[26] Xu W., Cai J., Mishra K.V., Cho M., Kruger A. Precise semidefinite programming formulation of atomic norm minimization for recovering d-dimensional (d ≤ 2) off-the-grid frequencies. Proceedings of the 2014 Information Theory and Applications Workshop (ITA).

[27] De Castro Y., Gamboa F., Henrion D., Lasserre J.B. (2019). Approximate optimal designs for multivariate polynomial regression. Ann. Stat..

[28] Semper S., Romer F. ADMM for ND Line Spectral Estimation Using Grid-free Compressive Sensing from Multiple Measurements with Applications to DOA Estimation. Proceedings of the IEEE International Conference on Acoustics, Speech and Signal Processing (ICASSP).

[29] Lee H.B., Wengrovitz M.S. (1990). Resolution threshold of beamspace MUSIC for two closely spaced emitters. IEEE Trans. Acoust. Speech Signal Process..

[30] Tan Z., Eldar Y.C., Nehorai A. (2014). Direction of Arrival Estimation Using Co-Prime Arrays: A Super Resolution Viewpoint. IEEE Trans. Signal Process..

[31] Zoltowski M.D., Kautz G.M., Silverstein S.D. (1993). Beamspace Root-MUSIC. IEEE Trans. Signal Process..

[32] Wagner M., Gerstoft P., Park Y. Gridless DOA Estimation via. Alternating Projections. Proceedings of the IEEE International Conference on Acoustics, Speech and Signal Processing (ICASSP).

[33] Anderson S. (1993). On Optimal dimension reduction for sensor array signal processing. Signal Process..

[34] Grant M., Boyd S. CVX: Matlab Software for Disciplined Convex Programming, version 2.0 beta. http://cvxr.com/cvx.

